# On current transients in MoS_2_ Field Effect Transistors

**DOI:** 10.1038/s41598-017-11930-6

**Published:** 2017-09-14

**Authors:** Massimo Macucci, Gerry Tambellini, Dmitry Ovchinnikov, Andras Kis, Giuseppe Iannaccone, Gianluca Fiori

**Affiliations:** 1 Dipartimento di Ingegneria dell’Informazione, University of Pisa, 56122 Pisa, Italy; 2 Institutes of Electrical engineering and Materials Science and Engineering, École Polytechnique Fédérale de Lausanne, CH-1015 Lausanne, Switzerland

## Abstract

We present an experimental investigation of slow transients in the gate and drain currents of MoS_2_-based transistors. We focus on the measurement of both the gate and drain currents and, from the comparative analysis of the current transients, we conclude that there are at least two independent trapping mechanisms: trapping of charges in the silicon oxide substrate, occurring with time constants of the order of tens of seconds and involving charge motion orthogonal to the MoS_2_ sheet, and trapping at the channel surface, which occurs with much longer time constants, in particular when the device is in a vacuum. We observe that the presence of such slow phenomena makes it very difficult to perform reliable low-frequency noise measurements, requiring a stable and repeatable steady-state bias point condition, and may explain the sometimes contradictory results that can be found in the literature about the dependence of the flicker noise power spectral density on gate bias.

## Introduction

In the last few years 2-D materials based on metal dichalcogenides have attracted increasing interest, because they share many of the properties of graphene, while having a nonzero bandgap, which makes them more suitable candidates for the implementation of switching devices for digital electronics. Among such 2-D materials, Molybdenum disulfide (MoS_2_) exhibits very interesting characteristic, because of its direct energy gap of 1.8–1.9 eV (bulk MoS_2_ has a smaller and indirect gap of 1.3 eV). This characteristic is of pivotal importance for the fabrication of devices for digital applications, since a large *I*_o*n*_/*I*_o*ff*_ ratio can be achieved. On the other hand, MoS_2_ has a mobility much smaller than graphene, up to only a few hundreds of cm^2^ V^−1^ s^−1^ ^1^. The actual mobility is strongly dependent also on the surface condition of the material, i.e. on the presence of adsorbates, in particular water or oxygen. Late *et al*.^2^ have investigated the effect of adsorbates on the hysteretic behavior and on current transients in MoS_2_ transistors, focusing, in particular, on the former. Further studies of the hysteretic behavior of MoS_2_ transistors have been performed by Li *et al*.^3^, Illarionov *et al*.^4^, and Guo *et al*.^5^.

Future implementations of these transistors, with a much thinner dielectric layer and with improved mobility, could possibly be exploited for particular low-power digital applications in which their intrinsically 2-D nature can represent an important advantage, such as in bendable electronics or in the case of 3-D integration.

We have studied the gate and drain current transients in non-passivated MoS_2_ transistors fabricated by means of mechanical exfoliation and scotch tape transfer onto a silicon dioxide substrate.

Our interest in the current transients in MoS_2_ devices has actually been triggered by the investigation of low-frequency noise, to which the same trapping and detrapping phenomena that are at the basis of the transients are expected to contribute significantly.

While trying to perform repeatable 1/*f* noise measurements, we realized that it was very hard, in particular if the devices were kept in a vacuum, to reach the steady-state bias condition that needs to be achieved before acquiring the time records for the evaluation of the power spectral density. In the presence of a slow exponential decay of the bias current and of significant hysteresis, the result of the measurement would be severely affected, not in terms of the frequency dependence of the power spectral density, which turns out to be 1/*f* anyway, if we consider the usual frequency range, extending down to about 10 Hz, but in terms of the dependence of the value of the noise power spectral density at a given frequency on the drain current and on the gate bias voltage (i.e. the most relevant information when investigating the origin of 1/*f* noise). In particular, in the presence of a strong hysteresis, it is extremely difficult to obtain repeatable gate bias conditions, since, for the same gate voltage different values of the drain current will be present, depending on the previous bias history.

In ref. 2 the variance observed in measurement results relative to MoS_2_ devices is attributed to their photosensitivity combined with the fluctuations of ambient illumination. Although this may be a concurring cause of the variance, it is not the only one, because we have observed fluctuations also with the devices kept in a completely dark environment, as in most of our measurements.

Such difficulties and slow transients may contribute to explain why the dependence of 1/*f* noise on the bias condition in MoS_2_ transistors has been investigated by several authors with results that appear to be often in contrast with one another.

In particular, Sangwan *et al*.^6^ argue that the normalized flicker noise (i.e. the noise power spectral density divided by the square of the current) scales with the inverse of the gate overdrive, thereby supporting an interpretation in terms of a bulk Hooge formula approach and a mobility fluctuation model, if one assumes that the carrier density in the channel is proportional to the gate overdrive.

In ref. 7 by Sharma *et al*. a somewhat opposite conclusion is reached, attributing flicker noise observed in single-layer MoS_2_ devices to the McWhorter model, i.e. mainly to carrier number fluctuation with some contribution from mobility modulation still due to trap occupancy fluctuation.

For multilayer devices, Na *et al*.^8^ argue for a mobility fluctuation, Hooge-like model, but reintroduce the carrier fluctuation model to explain the behavior in the presence of Al_2_0_3_ passivation.

Renteria *et al*. in ref. 9 state substantially the opposite for bi- and tri-layer, explaining the measured flicker noise in terms of carrier number fluctuations.

Kwon *et al*.^10^ present an analysis similar to that in ref. 6 for the case of multilayer devices.

Xie *et al*.^11^ argue that number fluctuation is the main source of flicker noise in bilayer MoS_2_ devices, with a gate voltage dependence of the normalized noise power spectral density that is however quite different from what reported by other authors.

Finally, in ref. 12 Wang *et al*. attribute the 1/*f* noise seen in few-layer MoS_2_ devices to a Hooge-like mechanism on the basis of the fact that they observe a proportionality of the noise power spectral density to the square of the drain current.

In the following we focus on the measurement of current transients in MoS_2_ transistors and on their interpretation in terms of charge trapping phenomena. The presence of extremely long time constants and of variable amounts of trapped charge makes a reliable estimation of the bias point dependence of low-frequency noise power spectral density quite challenging.

## Measurement Results and Interpretation

**Figure 1:**
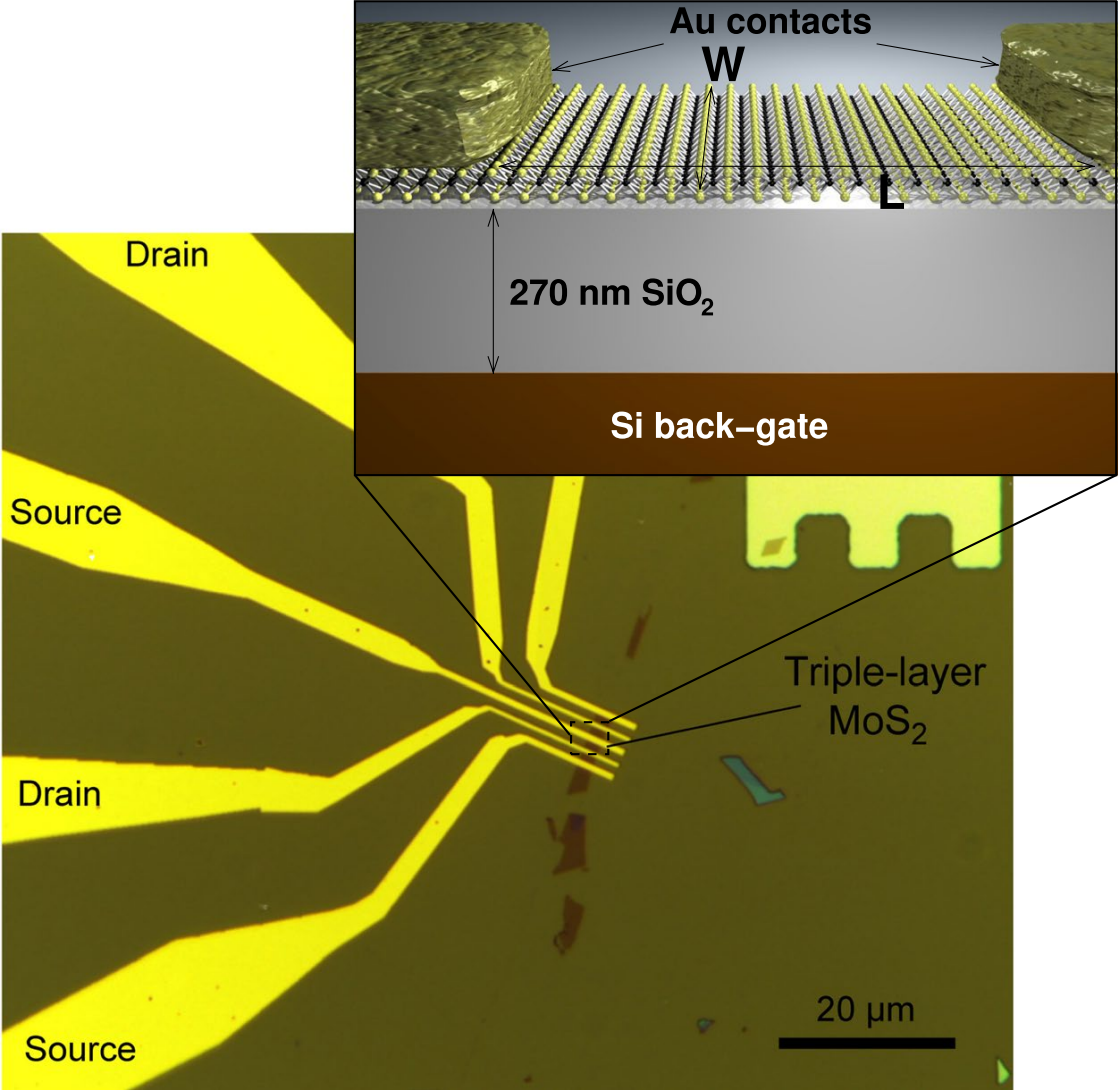
Micrograph of the MoS_2_ transistor on which of the current transient measurements have been performed. The inset contains a sketch of the device structure.

Single-layer and triple-layer devices have been fabricated with a process similar to that of previous reports^1^. Atomically thin MoS_2_ flakes were obtained with scotch-tape exfoliation of bulk MoS_2_ crystals (SPI supplies) on degenerately doped Si substrates covered with 270 nm thick thermally grown SiO_2_ for optimized contrast detection^13^. Contacts were fabricated with electron beam lithography using PMMA as resist, followed by evaporation of 90 nm of Au and liftoff in acetone. Furthermore, selected devices were annealed in Ar atmosphere at 200 °C to improve the contact resistance (see ref. 1). A silicon substrate was used as a global back gate for all measured devices. The channel of the device is *L* = 1.7 *μ*m long and *W* = 4.3 *μ*m wide, and consists of monolayer material. A micrograph of the device is shown in Fig. 1.

**Figure 2:**
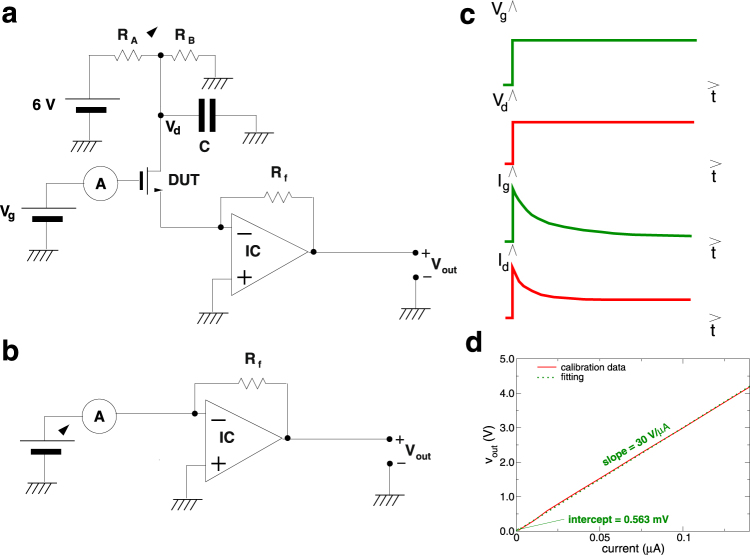
(**a**) Schematic of the setup for the measurement of the gate and channel current transients in MoS_2_ transistors. (**b**) Schematic of the setup for the calibration of the transresistive amplifier used for the amplification of the channel current. (**c**) Qualitative plot of the applied voltages and measured currents. (**d**) Example of calibration data obtained with a 30 MΩ feedback resistor.

Current transient measurements have been performed applying a voltage step to the drain and gate electrodes between zero and the target bias voltage, and monitoring the evolution in time of the currents flowing in the two electrodes (Fig. 2c). In particular, while in past studies^2, 5^ time evolution was investigated only for the drain current, we have acquired also the behavior of the gate current, which provides extremely relevant information about the nature of the transients.

We first report on the results of a measurement performed by applying a gate voltage of 20 V, with the circuit of Fig. 2a, where the DUT (Device Under Test) source is at virtual ground via the input of a transresistive amplifier and the drain is biased with 100 mV obtained by means of the resistive partition (*R*_*A*_, *R*_*B*_) of the output of a 6 V lead battery included in the same shielding as the amplifier. The capacitor *C* has the purpose of filtering the noise that could be introduced by the partition. The voltage measured at *V*_*out*_ is proportional to the current flowing out of the DUT source electrode through the value of the feedback resistor. In series with the gate we have a picoammeter (HP 4140B), to measure the gate current, and the voltage bias source.

For a precise evaluation of the current in the device channel, we perform a calibration step, with the circuit arrangement shown in Fig. 2b: by varying the value of the adjustable voltage source, we scan the input current *i*_in_ of the transresistive amplifier over a wide range, and then, from a linear fit of the measured *v*_out_ vs. *i*_in_, we get a good estimate of the actual value of the feedback resistor (from the slope) and of the voltage offset at the output of the amplifier (from the intercept at zero input current). An example of a calibration measurement, together with the associated linear fitting, is reported in Fig. 2d.

**Figure 3:**
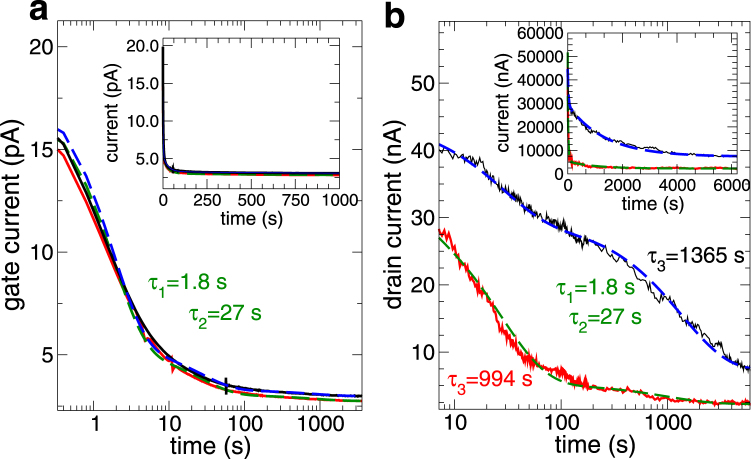
Plots of the gate current (left panel) and drain current (right panel) transients when the MoS_2_ transistor is in air (thick red curves) and in a vacuum (thin black curves). The dashed curves represent the fitting as a combination of two exponentials. The applied gate voltage is 20 V and the drain bias is 100 mV.

In Fig. 3 we report the measured transients of the gate (left panel) and channel (right panel) currents for the device, kept either in a vacuum (about 10^−4^ Torr) or in the air.

It is immediately apparent that in a vacuum the gate current and the channel current have a rather different behavior, while in air they are much more similar. Thus in a vacuum there seems to be a further trapping mechanism active, which affects only the channel current. Let us first discuss the gate current behavior: as already stated, it is independent of the presence of air, with a quick exponential decay, which can be associated with trapping of charge carriers in traps with relatively short characteristic times. The gate current is made up of two components: the conduction current and the displacement current. The conduction current is the result of leakage from the gate to the channel through the oxide and is substantially dependent only on the applied gate bias, while the displacement current is the consequence of charge moving orthogonally to the oxide layer, without actually reaching the gate. We interpret the asymptotic value of the gate current as the leakage (conduction) component, while the much larger time-dependent component can be seen as the displacement current resulting from the motion of electrons being injected from the MoS_2_ sheet into the dielectric substrate and then trapped in the proximity of the MoS_2_-silicon oxide interface.

This phenomenon had been excluded in ref. 2 on the basis of the fact that local breakdown of a thick oxide is unlikely; however for a charge carrier to be trapped in the dielectric layer in the proximity of the channel no insulator breakdown is indeed needed and, in particular, no conduction current to the gate is required: there will be only a displacement current resulting from the motion of the charge, shifting from the MoS_2_ sheet to a position slightly closer to the gate. In particular, from the Ramo-Shockley theorem^14, 15^ or from simple electrostatic considerations, we know that, for each charge *q*_*c*_ moving from the channel to a trap at a depth Δ*z* in the SiO_2_, a charge variation Δ*Q* = (Δ*z*/*t*_*ox*_)(−*q*_*c*_) is induced in the gate.

The interpretation of the current transient measured on the gate as associated with charge trapping in the oxide is further strengthened by the results obtained for the gate current transient occurring when the gate bias voltage is switched off, which exhibits an exponential behavior analogous to that of the switch-on transient. This excludes the hypothesis that the switch-on exponential could be due, for example, to a leakage current to the channel following, for some undetermined reason, the same behavior of the channel current.

**Figure 4:**
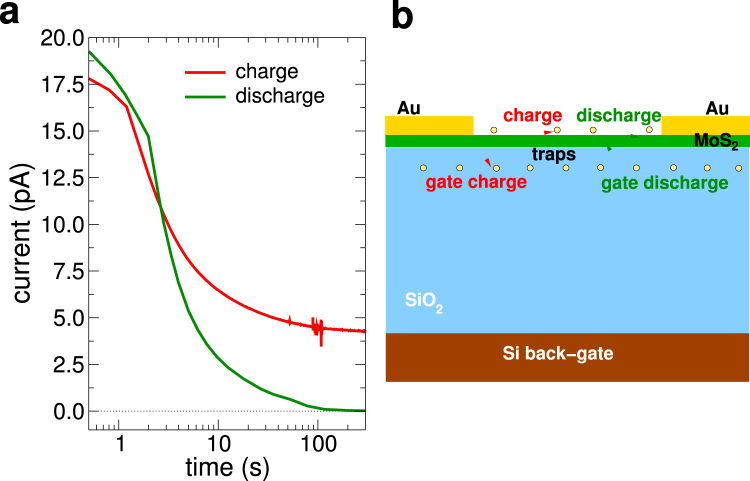
(**a**) Plot of the charging (red curve) and the discharging (green curve) transients for the gate current. (**b**) Sketch of the trapping processes.

In Fig. 4a we show both the switch-on (red curve) transient (which tends to saturate to the DC leakage current value, approximately 4.27 pA) and the switch-off (green curve) transient (which tends to zero), while in Fig. 4b, we plot a sketch of the underlying trapping processes that we propose in the following to explain the observed current transients. If we integrate over time both curves (after subtracting their asymptotic values), we obtain a similar total charge in the two transients, of about 1.5 × 10^−10^ C (*Q*_*C*_ and *Q*_*D*_, respectively, which correspond to the shaded area highlighted in Fig. 4a). In order to relate this charge with the actual charge being trapped, we must consider that the traps are located at a distance from the MoS_2_ layer that is at most of a few nanometers (on the basis of data that can be found in the literature for the Si/Si0_2_ interface^16–18^, since data for the MoS_2_/SiO_2_ interface are not available), thus a fraction of the total oxide thickness. This means that, considering a charge penetration of about 3 nm vs. an oxide thickness of 270 nm, the trapped charge will be almost two orders of magnitude larger than the charge measured in the gate transients, i.e. about 15 × 10^−9^ C. This corresponds to about 10^10^ electrons and, considering that the metal leads contact a number of MoS_2_ flakes, each with an average area of the order of a hundred square microns, the resulting density of trapped charge is around 10^15^ cm^−2^, which is consistent with the upper values reported in the literature^5, 19, 20^. It can actually be lower if we consider that charge trapping will occur also underneath the metal lines (whose area is larger than that of the contacted MoS_2_ flakes.

Let us now discuss the features of the drain (channel) current, reported in the right panel of Fig. 3: as already observed by Late *et al*.^2^, there is a significant difference between the time evolution of the current in air, and that in a vacuum. While in air the transient behavior is close to that of the gate current, in a vacuum the presence of a much longer time constant can be observed. We have tried to fit both the gate and the drain currents as a sum of exponentials plus a constant: *i*_*g*_(*t*) = *a* exp(−*t*/*τ*_1_) + *b* exp(−*t*/*τ*_2_) + *c* for the gate current and *i*_*d*_(*t*) = *d* exp(−*t*/*τ*_1_) + *e* exp(−*t*/*τ*_2_) + *f* exp(−*t*/*τ*_3_) + *g* for the drain current. The choice of two exponentials for the gate current and of three for the drain current has been made on the basis both of the physical argument that the trapping phenomenon leading to the gate displacement current must impose on the drain current an analogous transient (since the trapped charge will screen the electric field from the gate), with the same time constants and of the observation that this choice leads to the best fit. Accordingly, the fit has been performed for the gate current first, and then the one for the drain current has been completed assuming the two lowest time constant equal to those of the gate current. Overall, the choice of the number of time constants for the fit unavoidably involves some arbitrariness, also because of the critical nature of this type of fittings^21^.

For the gate transients we obtain, independent of the presence of air: *τ*_1_ = 1.8 s and *τ*_2_ = 27 s (*a* = 12.64 pA, *b* = 2.16 pA, *c* = 2.74 pA). For the drain current in air we get *τ*_3_ = 994 s (*d* = 18.61 nA, *e* = 27.8 nA, *f* = 3.03 nA, *g* = 2.27 nA), while in a vacuum we have *τ*_3_ = 1365 s (*d* = 0.12 nA, *e* = 15.1 nA, *f* = 22.1 nA, *g* = 7.32 nA). We propose an interpretation of these results in terms of the previously mentioned capture of charge carriers in oxide traps with the shorter time constants (those appearing only in the gate current), which lead to the transient in the gate current and to a modulation of the channel current with the same time constants.

In air the drain current exhibits only the small additional effect of another time constant (994 s), with a relatively small amplitude for the associated exponential. In vacuum, instead, the asymptotic value of the drain current is much larger and the long time constant (1365 s) has an amplitude greater by an order of magnitude than in air, which makes quite unpractical any measurement requiring a steady-state condition to be maintained over a long period of time, as in the case of low-frequency noise measurements.

A possible explanation of such a behavior can be constructed assuming that the longer time constant is associated with traps that are at the exposed surface of the MoS_2_ sheet and therefore do not involve a vertical motion of charge (in the direction orthogonal to the gate) which would register in the gate current. The presence of air is modifying these surface traps (possibly resulting from residual moisture or from molecules of other gases adsorbed on the surface of the 2-dimensional material). We point out that both trapping phenomena, the one associated with the short time constants and that associated with the long time constants, lead to a decrease of the channel current. For the shorter time constants trapping of negative charge in the oxide below the MoS_2_ channel is consistent with a decrease of the channel current (while it would not be consistent with alternative explanations for the gate current, such as additional charge entering the channel from the source and drain electrodes); for the longer time constants trapping at the surface of the MoS_2_ of charge that is already present in the channel (and therefore without contribution to the gate current) corresponds to a decrease of the mobile charge, and therefore of the current. The two proposed trapping processes are illustrated in Fig. 4b.

**Figure 5:**
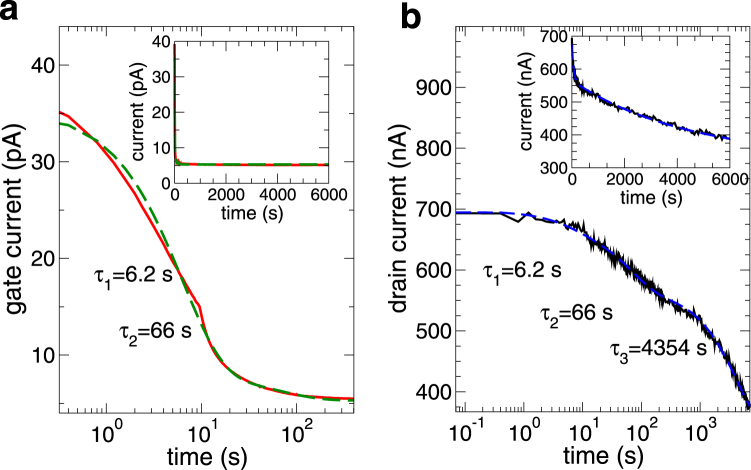
Plots of the gate current (left panel) and drain current (right panel) transients for the same MoS_2_ transistor as in Fig. 3, but for an applied gate voltage of 30 V and drain bias of 250 mV. Measurements have been performed in a vacuum.

In Fig. 5 we show another transient for the same transistor as in Fig. 3, but with a different gate voltage *V*_*G*_ = 30 V and drain bias *V*_*D*_ = 250 mV. These measurements were performed in a vacuum. For the gate current we have *τ*_1_ = 6.2 s and *τ*_2_ = 66 s (*a* = 27.4 pA, *b* = 2.74 pA, *c* = 5.3 pA), while for the drain current we get *τ*_3_ = 4354 s (*d* = 328 nA, *e* = 28.6 nA, *f* = 102.65 nA, *g* = 237.11 nA) We notice that the overall behavior is analogous to that for the previous bias point, although the time constants are somewhat different. Such a difference can be, at least in part, due to the sensitivity of the exponential fitting procedure to small variations. Although it is thus difficult to provide exact numbers, we can conclude that the trapping phenomena associated with the gate current have time constants of the order of seconds or tens of seconds, while in vacuum the drain current exhibits also the effect of a time constant of the order of thousands of seconds.

**Figure 6:**
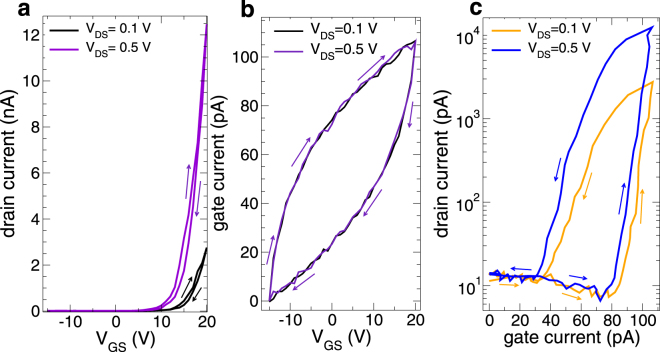
(**a**) Plot of the transfer characteristic of an MoS_2_ transistor for *V*_*GS*_ varying between −15 and +20 V with a forward and a backward scan. (**b**) Gate current vs. gate voltage for the same scans as in (**a**); (**c**) drain current vs. gate current for the same scans as in (**a**). In all panels data for drain bias values of 0.1 and 0.5 V are reported.

In Fig. 6 we report the hysteretic behavior observed when acquiring the device DC characteristics over a time of a few tens of seconds, for constant values of the drain-source bias of 0.1 and 0.5 V. Figure 6a reports the drain current vs. gate voltage for a variation of the gate voltage, forward and back, from −15 to +20 V. We see a moderate hysteresis on the drain current, while the gate current exhibits a very significant hysteresis, as shown in Fig. 6b, which is the result of the charging and discharging of the traps in the oxide.

In Fig. 6c, we report instead the drain current vs. the gate current, with the same values of the drain current corresponding, during the forward scan, to much larger values of the gate current than in the backward scan, as a result of the gate charging process.

## Conclusion

The comparative analysis of the time-dependent behavior of the gate and channel currents in MoS_2_ field effect transistors has allowed us to assess the presence of two distinct phenomena, dominated by different time constant: a faster process in which charges move from the channel to traps in the oxide, along a path that determines the appearance of displacement current in the gate, as well as an associated modulation in the drain current; a slower process in which carriers are captured by traps at the surface of the channel, without a significant shift in the direction of the gate (and therefore without an influence on the gate current). This latter process has a much stronger effect in a vacuum, arguably because the associated time constant is reduced by the presence of air or because adsorbates from air act as much more efficient and faster traps. The resulting long transients make it very difficult to perform reliable noise measurements at low frequencies, and may explain the strong variability of results observed in experimental studies of 1/*f* noise in MoS_2_ devices.
